# Practice pattern of aerosol therapy among patients undergoing mechanical ventilation in mainland China: A web-based survey involving 447 hospitals

**DOI:** 10.1371/journal.pone.0221577

**Published:** 2019-08-29

**Authors:** Zhongheng Zhang, Peifeng Xu, Qiang Fang, Penglin Ma, Huiling Lin, Jim B. Fink, Zongan Liang, Rongchang Chen, Huiqing Ge, on behalf of the China Union of Respiratory Care (CURC)

**Affiliations:** 1 Department of emergency medicine, Sir Run Run Shaw Hospital, Zhejiang University School of Medicine, Hangzhou, China; 2 Department of Respiratory Care, Sir Run Run Shaw Hospital, Zhejiang University School of Medicine, Hangzhou, China; 3 Department of critical care medicine, First hospital, Zhejiang University School of Medicine, Hangzhou, China; 4 Department of Critical Care Medicine, Eighth Medical Center of PLA General Hospital, Beijing, China; 5 Department of Respiratory Therapy, Chang Gung University, Taoyuan City, Taiwan; 6 Aerogen Pharma Corp., San Mateo, California, United States of America; 7 Department of Respiratory and Critical Care Medicine, West China Medical Center, Sichuan University, China; 8 Guangzhou Institute of Respiratory Diseases, Guangzhou, China; San Gerardo Hospital, ITALY

## Abstract

**Background and objective:**

Aerosol therapies are widely used for mechanically ventilated patients. However, the practice pattern of aerosol therapy in mainland China remains unknown. This study aimed to determine the current practice of aerosol therapy in mainland China.

**Methods:**

A web-based survey was conducted by the China Union of Respiratory Care (CURC) from August 2018 to January 2019. The survey was disseminated via Email or WeChat to members of CURC. A questionnaire comprising 16 questions related to hospital information and 12 questions related to the practice of aerosol therapy. Latent class analysis was employed to identify the distinct classes of aerosol therapy practice.

**Main results:**

A total of 693 valid questionnaires were returned by respiratory care practitioners from 447 hospitals. Most of the practitioners used aerosol therapy for both invasive mechanical ventilation (90.8%) and non-invasive mechanical ventilation (91.3%). Practitioners from tertiary care centers were more likely to use aerosol therapy compared with those from non-tertiary care centers (91.9% vs. 85.4%, respectively; p = 0.035). The most commonly used drugs for aerosol therapy were bronchodilators (64.8%) followed by mucolytic agents (44.2%), topical corticosteroids (43.4%) and antibiotics (16.5%). The ultrasonic nebulizer (48.3%) was the most commonly used followed by the jet nebulizer (39.2%), the metered dose inhaler (15.4%) and the vibrating mesh nebulizer (14.6%). Six latent classes were identified via latent class analysis. Class 1 was characterized by the aggressive use of aerosol therapy without a standard protocol, while class 3 was characterized by the absence of aerosol therapy.

**Conclusions:**

Substantial heterogeneity among institutions with regard to the use of aerosol therapy was noted. The implementation of aerosol therapy during mechanical ventilation was inconsistent in light of recent practice guidelines. Additional efforts by the CURC to improve the implementation of aerosol therapy in mainland China are warranted.

## Introduction

Aerosol therapy, widely used for patients undergoing mechanical ventilation (MV), has the ability to confer positive effects via multiple mechanisms [1]. Nebulized drugs can be directly delivered to the airways and the lung parenchyma, thereby increasing the concentrations of these drugs locally and lowering the potential for systemic toxicities [2]. Increased local concentrations of nebulized antibiotics and rapid bacterial killing were noted in the lungs following the administration of these drugs during MV [3,4]. Nevertheless, there is limited evidence regarding the use of nebulized antibiotics in ventilated patients [5]. Although the effectiveness of aerosol therapy with regard to patient-centered outcomes is controversial [6–8], an international survey showed that most physicians (99%) support the use of this therapy during both invasive MV (IMV) and non-invasive MV (NIMV) [9]. A major obstacle for drug delivery via nebulization is the low delivery efficiency of the nebulizers [10]; the majority of the aerosol is generally deposited into the ventilator circuit and the endotracheal tube. The jet nebulizers, in particular, are limited by their high residual volume. A variety of technologies and techniques have been developed to address this problem. For example, the vibrating mesh and ultrasonic nebulizers were shown to increase the delivered dose to the patient when compared with the jet nebulizer [11,12]. Furthermore, ventilator settings, respiratory parameters and nebulizer position can influence drug delivery [13,14].

The majority of the previous studies were conducted in vitro and in animal models; in vivo studies using radiolabeling are limited. Thus, there is a lack of randomized controlled trials to determine the most appropriate technique or ventilator setting in terms of the clinical outcomes measured [15]. The practice patterns of aerosol therapy have evolved over time and varied substantially in different institutions. The jet, ultrasonic and vibrating mesh nebulizers were used in 55%, 44% and 14% respondents in previous surveys [9]. China is a large country with substantial variances in the practice of aerosol therapy during MV [16]. However, sufficient information about the use of aerosol therapy by respiratory care practitioners and intensivists in China is lacking. Thus, the present study aimed to determine the current practices involved in aerosol therapy across mainland China using latent class analysis to categorize institutions into a number of classes with distinct practice patterns. We believe that this information will aid in developing better policies and educational programs.

## Methods

### Survey questionnaire

This study was approved by the institutional review board of the Sir Run Run Shaw hospital (Hangzhou, China) (20151201–17). Informed consent was obtained from all the participants. A web-based survey was conducted from August 2018 to January 2019. The survey was disseminated via Email or WeChat to 2000 members via the platform of the China Union of Respiratory Care (CURC). Deidentified dataset is available as supporting information (S1 Dataset). The aim of the CURC, which comprises respiratory care practitioners from mainland China, is to improve the quality of the respiratory care and conduct clinical researches. Members of CURC included respiratory care practitioners from mainland China. Only one member was invited from one department (e.g. one hospital may have several departments such as medical and surgical ICUs in which aerosol therapy is used. They could have different practice patterns of aerosol therapy). The questionnaire was explained in a detailed manner by the organizer of the meeting. The questions on aerosol therapy included the following topics: type of drug delivered during IMV or NIMV, type of nebulizer used, type of jet nebulizer used, position of the nebulizer in the case of small-volume nebulizers, position and use of a spacer chamber with a metered-dose inhaler (MDI), frequency of changing the filters in a ventilator circuit, ventilator setting during aerosol therapy, positive end-expiratory pressure (PEEP) level during nebulization, ventilator mode during nebulization, use of a nebulization protocol, and assessment of the effectiveness of the nebulization. The full questionnaire can be accessed at the following web link: http://client.rup-china.com/icu/wx/?from=singlemessage&isappinstalled=0#/?time=1549868372. A translated version of this questionnaire is enclosed as a supporting file (S1 Questionnaire).

Tertiary care refers to the specialized consultative healthcare provided to inpatients and patients referred from primary and secondary healthcare centers for advanced medical investigation and treatment. The respondents in this study were categorized into two groups, those from tertiary care hospitals and those from non-tertiary care hospitals.

### Statistical analysis

Categorical variables were expressed as numbers and percentages. Differences between tertiary and non-tertiary care centers were compared using the Chi-square or Fisher’s exact test as appropriate. Continuous data were expressed as median and interquartile range and were compared using non-parametric tests between groups [17]. Statistical descriptions and bivariate inferences were performed using the *CBCgrps* package [18]. All statistical analyses were performed using RStudio (Version 1.1.463).

Latent class analysis (LCA) was performed to identify the classes of hospitals with distinct practice patterns of aerosol therapy [19]. The rationale for using LCA was that this technique allows for the modeling of distinct practice patterns of aerosol therapy using individual questions in the form of a questionnaire. The identification of distinct practice patterns may help formulate specific training programs and policies for different hospitals. LCA models with one to seven classes were fitted using response variables, which included all of the 12 questions related to aerosol therapy. Some questions with more than two response items were dummified resulting in k-1 variables (k, number of response items). The fitness of each model was then compared (how well each model described the underlying data). Entropy describes the dispersion (or concentration) in a probability mass function and is expressed as a value ranging from 0 to 1. An entropy value approaching 0 indicates that the observations were well categorized into the latent classes, whereas a value approaching 1 indicates a model that categorizes the observations poorly. Thus, among all the fitted models, we intended to choose the one with the lowest entropy value. Other statistics such as Bayesian information criterion (BIC), corrected Akaike information criterion (cAIC), and adjusted BIC (aBIC) were also reported. Lower BIC and AIC values indicate a better-fitted model [20–22].

## Results

### General description of the practice of aerosol therapy

A total number of 693 completed questionnaires (returning rate 693/2000 = 34.7%) were returned by the respiratory care practitioners from 447 hospitals. The samples were distributed across all the provinces of mainland China. Fifty-seven respondents (9.2%) reported never using aerosol therapy during MV, 15.4% exclusively used jet nebulizers, 26.3% exclusively used ultrasonic nebulizers, and 2.0% exclusively used metered dose inhalers (MDIs). Practitioners from tertiary care centers were more likely to use aerosol therapy when compared to those from non-tertiary care centers (91.9% vs. 85.4%, respectively; p = 0.035).

The most commonly used drugs for aerosol therapy were bronchodilators (64.8%) followed by mucolytic agents (44.2%), topical corticosteroids (43.4%) and antibiotics (16.5%). Interestingly, practitioners from tertiary care centers were more likely to use mucolytic agents (p = 0.03) and topical corticosteroids (p = 0.01) when compared to those from the non-tertiary centers. Ultrasonic nebulizers (48.3%) were most commonly used followed by jet nebulizers (39.2%), metered-dose inhalers (15.4%), and vibrating mesh nebulizers (14.6%). Jet nebulizers were used with an external gas source by 27.1% of the practitioners, while 20.2% used other external nebulizer pumps; about 28.6% reported the use of ventilator- integrated systems. Metered-dose inhalers were used via an inhalation chamber (344 respondents; 49.6%) placed within the circuit or directly into the tracheal tube after disconnecting the patient (111 respondents; 16%); 11.4% of the practitioners never think about this problem. The most common position of nebulizer for small-volume nebulizer was placed at the inspiratory limb near the Y-piece (39.8%). In terms of changing the ventilator settings, 40.8% of the respondents reported not changing the setting during aerosol therapy, whereas 11.3% and 17% of the respondents reported increasing the tidal volume and the inspiratory time, respectively.

Most practitioners assessed the effectiveness of the aerosol therapy by observing the waveform (45.6%), relying on the auscultation of the pulmonary sound (33.8%), and observing the breathing by physical examination (31.2%). Only 9.4% responders reported not assessing the effectiveness of the therapy (Table 1).

**Table 1:** Comparing Aerosol therapies between tertiary and non-tertiary care hospitals.

Variables	Overall (n = 693)	Non-tertiary care (n = 123)	Tertiary care center (n = 570)	p
Nebulization for IMV, n (%)	629 (90.8)	105 (85.4)	524 (91.9)	0.035
Nebulization for NIMV, n (%)	633 (91.3)	108 (87.8)	525 (92.1)	0.173
Drugs for IMV nebulization				
Bronchodilators, n (%)	449 (64.8)	78 (63.4)	371 (65.1)	0.804
Antibiotics, n (%)	114 (16.5)	18 (14.6)	96 (16.8)	0.642
mucolytic agent, n (%)	306 (44.2)	43 (35.0)	263 (46.1)	0.030
Topical corticosteroids, n (%)	301 (43.4)	40 (32.5)	261 (45.8)	0.010
Systemic corticosteroids, n (%)	59 (8.5)	9 (7.3)	50 (8.8)	0.729
Others, n (%)	14 (2.0)	1 (0.8)	13 (2.3)	0.486
No. of drug types used in aerosol therapy in a department (median [IQR])	3.00 [2.00, 4.00]	2.00 [1.00, 4.00]	3.00 [2.00, 4.00]	0.019
Nebulizer type				
Ultrasonic nebulizer (%)	335 (48.3)	60 (48.8)	275 (48.2)	0.993
Jet nebulizer, n (%)	272 (39.2)	40 (32.5)	232 (40.7)	0.113
vibrating-mesh nebulizer, n (%)	101 (14.6)	12 (9.8)	89 (15.6)	0.126
Metered dose inhaler, n (%)	107 (15.4)	21 (17.1)	86 (15.1)	0.678
Others, n (%)	24 (3.5)	2 (1.6)	22 (3.9)	0.339
No. of nebulizer type used in a department (median [IQR])	2.00 [2.00, 3.00]	2.00 [1.00, 3.00]	2.00 [2.00, 3.00]	0.146
Jet nebulizer implementation				
External gas source, n (%)	188 (27.1)	39 (31.7)	149 (26.1)	0.251
External nebulizer pump, n (%)	140 (20.2)	25 (20.3)	115 (20.2)	1.000
Nebulizer within ventilator, n (%)	198 (28.6)	23 (18.7)	175 (30.7)	0.010
Others, n (%)	9 (1.3)	0 (0.0)	9 (1.6)	0.335
No. of jet nebulizer implementations used in a department (median [IQR])	2.00 [2.00, 2.00]	2.00 [1.00, 2.00]	2.00 [2.00, 2.00]	0.060
Position of nebulizer for small-volume nebulizer*				
Inspiratory limb near Y-piece, n (%)	276 (39.8)	47 (38.2)	229 (40.2)	0.763
Humidifier proximal to ventilator, n (%)	126 (18.2)	22 (17.9)	104 (18.2)	1.000
Humidifier proximal to patient, n (%)	125 (18.0)	17 (13.8)	108 (18.9)	0.226
Position of nebulizer for metered dose nebulization*				
Inspiratory limb near Y-piece, n (%)	298 (43.0)	49 (39.8)	249 (43.7)	0.496
Humidifier proximal to ventilator, n (%)	115 (16.6)	18 (14.6)	97 (17.0)	0.610
Humidifier proximal to patient, n (%)	104 (15.0)	16 (13.0)	88 (15.4)	0.586
Use of holding chambers / spacers for metered dose nebulization				
Yes, n (%)	344 (49.6)	58 (47.2)	286 (50.2)	0.611
No, n (%)	111 (16.0)	21 (17.1)	90 (15.8)	0.829
Never use, n (%)	79 (11.4)	8 (6.5)	71 (12.5)	0.084
How often did your institution change the filter at expiratory circuit				
Every time after nebulization, n (%)	196 (28.3)	39 (31.7)	157 (27.5)	0.413
Once daily, n (%)	141 (20.3)	19 (15.4)	122 (21.4)	0.172
Twice a week, n (%)	74 (10.7)	13 (10.6)	61 (10.7)	1.000
Once a week, n (%)	100 (14.4)	13 (10.6)	87 (15.3)	0.229
More than once a week, n (%)	24 (3.5)	3 (2.4)	21 (3.7)	0.680
Change ventilator parameters during nebulization				
Never change, n (%)	283 (40.8)	47 (38.2)	236 (41.4)	0.581
Increase PEEP, n (%)	121 (17.5)	21 (17.1)	100 (17.5)	1.000
Decrease inspiratory flow, n (%)	90 (13.0)	12 (9.8)	78 (13.7)	0.304
Use constant inspiratory flow, n (%)	101 (14.6)	16 (13.0)	85 (14.9)	0.688
Increase inspiratory time, n (%)	118 (17.0)	16 (13.0)	102 (17.9)	0.240
Use inspiratory pause, n (%)	58 (8.4)	11 (8.9)	47 (8.2)	0.941
Increase tidal volume, n (%)	78 (11.3)	8 (6.5)	70 (12.3)	0.093
Stop heated humidifier, n (%)	64 (9.2)	7 (5.7)	57 (10.0)	0.185
Place filter on expiratory circuit, n (%)	76 (11.0)	6 (4.9)	70 (12.3)	0.026
Sedation to avoid dyssynchrony, n (%)	40 (5.8)	7 (5.7)	33 (5.8)	1.000
Use base flow rate, n (%)	24 (3.5)	3 (2.4)	21 (3.7)	0.680
Others, n (%)	7 (1.0)	0 (0.0)	7 (1.2)	0.460
No. of MV parameter changes (median [IQR])	2.00 [2.00, 4.00]	2.00 [1.00, 4.00]	2.00 [2.00, 4.00]	0.032
PEEP level during nebulization (cmH2O)				
0, n (%)	61 (8.8)	10 (8.1)	51 (8.9)	0.909
1–5, n (%)	242 (34.9)	45 (36.6)	197 (34.6)	0.747
6–10, n (%)	106 (15.3)	19 (15.4)	87 (15.3)	1.000
>10, n (%)	11 (1.6)	0 (0.0)	11 (1.9)	0.248
PEEP not changed, n (%)	52 (7.5)	6 (4.9)	46 (8.1)	0.303
Mode during nebulization				
Pressure control, n (%)	231 (33.3)	39 (31.7)	192 (33.7)	0.752
Pressure support, n (%)	191 (27.6)	31 (25.2)	160 (28.1)	0.593
Volume control, n (%)	169 (24.4)	28 (22.8)	141 (24.7)	0.729
SIMV, n (%)	149 (21.5)	24 (19.5)	125 (21.9)	0.638
High-frequency ventilation, n (%)	40 (5.8)	3 (2.4)	37 (6.5)	0.125
Others, n (%)	19 (2.7)	2 (1.6)	17 (3.0)	0.595
No. of ventilation mode (median [IQR])	2.00 [1.00, 2.00]	2.00 [1.00, 2.00]	2.00 [1.00, 3.00]	0.291
Nebulization protocol, n (%)	290 (41.8)	56 (45.5)	234 (41.1)	0.417
How to assess the effectiveness of nebulization				
Waveform, n (%)	316 (45.6)	53 (43.1)	263 (46.1)	0.606
Breathing sound, n (%)	234 (33.8)	35 (28.5)	199 (34.9)	0.205
Inspection of breathing appearance, n (%)	216 (31.2)	38 (30.9)	178 (31.2)	1.000
Not assess routinely, n (%)	65 (9.4)	11 (8.9)	54 (9.5)	0.990
Others, n (%)	19 (2.7)	3 (2.4)	16 (2.8)	1.000
No. of method to assess nebulization effectiveness (median [IQR])	2.00 [1.00, 3.00]	2.00 [1.00, 3.00]	2.00 [1.00, 3.00]	0.319
Bed No. (median [IQR])	1500 [1000, 2227]	876 [500, 1890]	1600 [1000, 2447]	<0.001
Overall evaluation of nebulization, n (%)				0.024
No response	2 (0.3)	2 (1.6)	0 (0.0)	
Very good	459 (66.2)	78 (63.4)	381 (66.8)	
Neutral	217 (31.3)	42 (34.1)	175 (30.7)	
Unsatisfactory	12 (1.7)	1 (0.8)	11 (1.9)	
Poor	3 (0.4)	0 (0.0)	3 (0.5)	
Previous survey experience of this type, n (%)				0.010
No response	2 (0.3)	2 (1.6)	0 (0.0)	
Yes	228 (32.9)	40 (32.5)	188 (33.0)	
No	463 (66.8)	81 (65.9)	382 (67.0)	

Note

* these percentages did not sum to 100 within a column because there were some non-respondents.

Abbreviations: MV: mechanical ventilation; SIMV: simultaneous intermittent mechanical ventilation; PEEP: positive end expiratory pressure; IQR: interquartile range; NIMV: non-invasive mechanical ventilation.

### Latent class analysis

**Fig 1:**
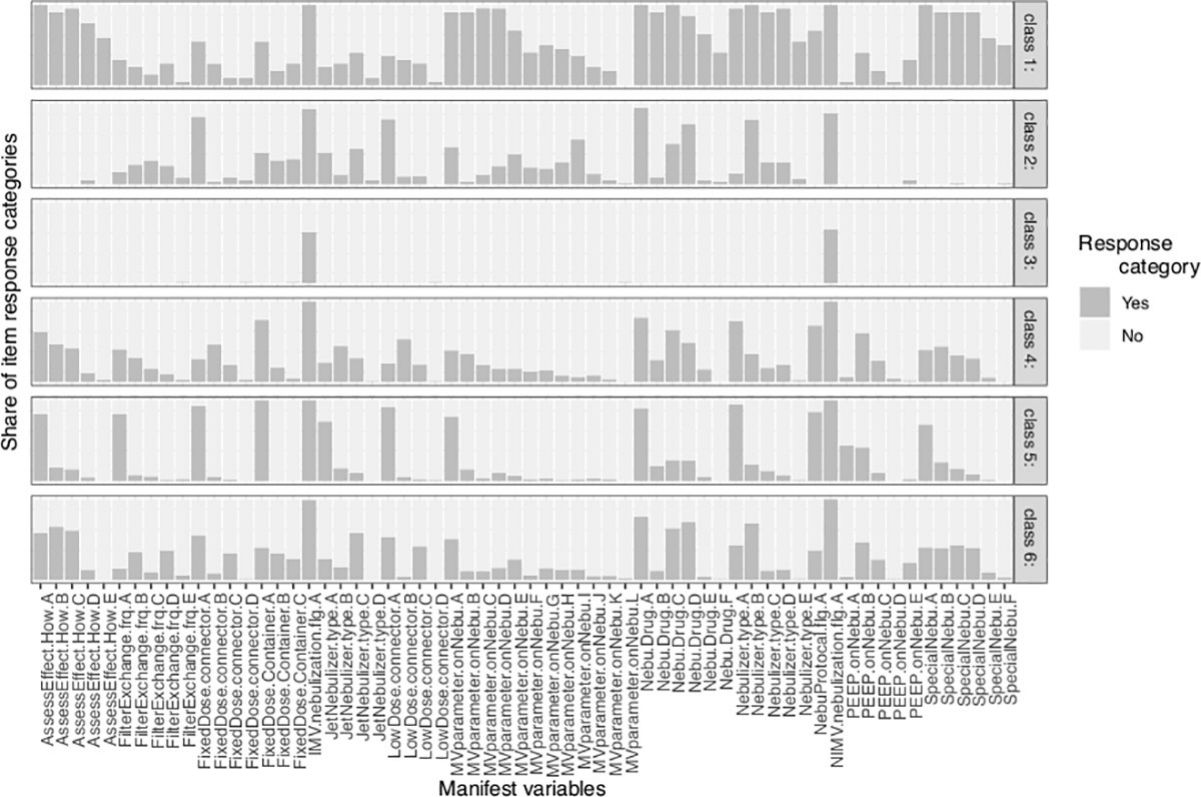
Characteristics of the latent classes. All questions related to the aerosol therapy are labeled on the horizontal axis. Each question was followed by an answer annotated by uppercase letters such as A, B, C, D and E. Annotations: how to assess the effectiveness of aerosol therapy (AssessEffect.How): A: Waveform B: Breathing sound C: Inspection of breathing appearance D: Not assess routinely E: others Filter exchange frequency (FilterExchange.frq) A: Every time after nebulization B: Once daily C: Twice a week D: Once a week E: More than once a week Nebulizer position for Metered dose nebulization (FixedDose.connector) A: Inspiratory limb near Y-piece B: Humidifier proximal to ventilator C: Humidifier proximal to patient D: others Use of holding chambers / spacers for metered dose nebulization (FixedDose.Container) A: yes B: no C: never use Use of aerosol therapy for invasive mechanical ventilation (IMV.nebulization.flg) A: Yes Type of jet nebulizer (JetNebulizer.type) A: external gas source B: external nebulizer pump C: nebulizer within ventilator D: others Nebulizer position for small-volume nebulizer (LowDose.connector) A: Inspiratory limb near Y-piece B: Humidifier proximal to ventilator C: Humidifier proximal to patient D: others Change of MV setting during nebulization (MVparameter.onNebu) A: Never change B: Increase PEEP C: Decrease inspiratory flow D: Use constant inspiratory flow E: Increase inspiratory time F: Use inspiratory pause G: Increase tidal volume H: Stop heated humidifier I: Place filter on expiratory circuit J: Sedation to avoid dyssynchrony K: Use base flow rate L: Others Type of nebulization drug (Nebu.Drug) A: bronchodilators B: antibiotics C: mucolytic agent D: topical steroids E: systemic steroids F: others Type of nebulizer (Nebulizer.type) A: ultrasonic nebulizer B: jet nebulizer C: vibrating mesh nebulizer D: metered dose E: others Use of protocol for aerosol therapy (NebuProtocal.flg) A: yes Use of nebulization during noninvasive mechanical ventilation (NIMV.nebulization.flg) A: yes PEEP level during aerosol therapy (PEEP.onNebu) A: 0 B: 1–5 C: 6–10 D: >10 E: PEEP not changed Model of ventilator setting during aerosol therapy (SpecialNebu) A: Pressure control B: Pressure support C: volume control D: SIMV E: High-frequency ventilation F: others.

Table 2 illustrates the statistics for choosing the most appropriate number of classes. The 6-class model showed the lowest entropy (0.92) and the lowest values in BIC (30125.84). aBIC demonstrated a continuous decrease from the 6-class to the 7-class model, albeit marginal. Furthermore, the increase in cAIC values from the 6-class model to the 7-class model was markedly greater than that from the 5-class model to the 6-class model (163.4 vs. 2.6, respectively). Thus, the 6-class model was considered as the best fit model. Class 1 was characterized by heterogeneous practice in each item (e.g. aggressive use of aerosol therapy without standard). For example, all answers to the question “how to assess the effectiveness of nebulization” were marked as yes (Fig 1). All drugs were used during ventilation. Class 3 was characterized by not using aerosol therapy; thus, most questions related to aerosol therapy were left unanswered. Class 5 was characterized by the uniform practice of aerosol therapy that each question had one choice, for example, most respondents in class 5 assess the effectiveness of nebulization by examining the waveform; and most of them used an ultrasonic nebulizer. This could be explained by the use of a standard protocol in these centers.

**Table 2:** Choosing the best number of classes for the latent class analysis.

log-likelihood	BIC	aBIC	cAIC	likelihood-ratio	Entropy	No. of classes
-15521.13	31859.89	31463.00	31984.89	23285.36	1.00	2
-14930.47	31090.66	30493.73	31278.66	22104.04	0.93	3
-14489.70	30621.20	29824.23	30872.20	21222.49	0.96	4
-14066.13	30186.14	29189.14	30500.14	20375.35	0.98	5
-13829.94	30125.84	28928.80	30502.84	19902.96	0.92	6
-13674.09	30226.23	28829.16	30666.23	19591.27	0.95	7

Abbreviations: BIC: Bayesian information criterion; cAIC: corrected Akaike information criterion; aBIC: adjusted Bayesian information criterion.

While class 4 and 5 were characterized by high proportion of tertiary care centers, class 1 and 3 were more likely from non-tertiary care centers (Table 3). Class 4 was characterized by larger number of hospital bed than class 3. Class 1 and 3 were from high-income regions, whereas class 5 was more likely to be in low income provinces.

**Table 3:** Characteristics of hospitals by latent classes.

	Class 1 (n = 99)	Class 2 (n = 181)	Class 3 (n = 158)	Class 4 (n = 66)	Class 5 (n = 22)	Class 6 (n = 167)	p
Tertiary care, n (%)	78 (78.8)	144 (79.6)	122 (77.2)	61 (92.4)	20 (90.9)	145 (86.8)	0.027
Bed No. (median [IQR])	1584 [1000, 2458]	1400 [996, 2000]	1200 [800, 2000]	2000 [1068, 2993]	1288 [1000, 2430]	1500 [861, 2474]	0.001
Provinces categorized by GDP (%)							<0.001
High	65 (65.7)	111 (61.3)	104 (65.8)	21 (31.8)	12 (54.5)	86 (51.5)	
Moderate	15 (15.2)	38 (21.0)	26 (16.5)	30 (45.5)	2 (9.1)	50 (29.9)	
Low	19 (19.2)	32 (17.7)	28 (17.7)	15 (22.7)	8 (36.4)	31 (18.6)	

Abbreviations: GDP: gross domestic product; IQR: interquartile range.

## Discussion

The present study demonstrates that the practice patterns of aerosol therapy varied substantially in mainland China. While some respondents believed that patients supported by ventilators cannot be treated with aerosol therapy, most respiratory care practitioners preferred to use this therapy for both IMV and NIMV. The most commonly used devices included jet, ultrasonic, and vibrating mesh nebulizers. There was a disparity between the tertiary and non-tertiary care centers with regard to the use of aerosol drugs. The practice pattern in this study was categorized into six latent classes. While class 1 was characterized by the aggressive use of aerosol therapy without a standard protocol, class 3 included respondents who did not use aerosol therapy. The clinical implication of the latent classes was to target hospitals that could benefit from the establishment of a practice protocol to standardize the implementation of the aerosol therapy. Different training programs can be implemented according to the distinct practice patterns of aerosol therapy. In the class 1 model, the practitioners were familiar with all the components of aerosol therapy, but they lacked knowledge about the effectiveness of each type of component; thus, the training program can focus on the indications of each component. Conversely, for institutions in the class 3 model, the training program should focus more on the technical details of each aspect of aerosol therapy.

Most of the results in the current study are consistent with that of an international survey conducted by the REVA (« Re´seau Europe´en » de recherche en Ventilation Artificielle) research network [9]. For example, both studies reported that bronchodilators and steroids were the most commonly used drugs for nebulization during MV. However, there are some distinctive characteristics with regard to the practice of aerosol therapy. The proportion of respondents who never changed the ventilator setting during nebulization was 77% in the international survey and 40% in the current study. This discrepancy may be attributed to the fact that jet nebulizers are not as widely used as ultrasonic and vibrating mesh nebulizers in Europe. The ultrasonic and vibrating mesh nebulizers do not add flow to the circuit and do not require changes in ventilator settings during therapy; on the other hand, ventilator settings need to be changed for the jet nebulizer. The current study was conducted six years after the REVA study, and there is emerging evidence of the importance of ventilator settings during aerosol therapy[23,24]. Furthermore, some clinical practice guidelines have been issued over the past few years [25]. Nebulization of antimicrobial agents was frequently performed, and ventilator-associated tracheobronchitis was one of the most common indications for this procedure [26]. Consistent with our study, inadequate practices were widely encountered, independent of the level of experience with the technique (e.g. direct tracheal instillation was considered for drug prescription in the majority of the ICUs) [27]. The jet nebulizer was most commonly used for antimicrobial agents as reported in a recent global survey [28]. However, the present study focused on all nebulization agents and found that the ultrasonic nebulizer was the most commonly used, followed by the jet nebulizer.

In the international survey, 65% respondents reported the integration of jet nebulization in the ventilator systems when compared to 28.6% in the current study. Almost 47.3% of the practitioners who used jet nebulizer utilized an external gas source in this study. Additional airflow affects ventilator pressure and flow, resulting in unstable ventilation. When the additional gas flow is used to operate the jet nebulizer during MV, the ventilator settings and alarms should be adjusted for patient safety [29]. In the current study, 5.8% respondents reported the need to increase sedation in order to improve ventilator-patient synchronization. Previous studies have shown that ventilation mode, breathing parameters, heat and humidity, gas density, and artificial airways influence aerosol delivery to critically ill subjects[12,14,30]. About 40.8% respondents reported never changing the ventilator settings during aerosol therapy; this included 33.9% and 20.8% of those who use the jet nebulizer and vibrating mesh nebulizer, respectively. Alternatively, 17.5% of respondents increased the PEEP, 13% decreased the inspiration flow, and 11.3% increased the tidal volume. Although a large tidal volume (VT) is associated with increased aerosol drug delivery during MV, it is essential to note that it can induce volutrauma and should not be used to improve the delivery efficiency of aerosol devices [31]. Increasing the inspiratory time leads to an increase in aerosol delivery; however, it is important to monitor the degree of intrinsic PEEP and exercise caution with this practice because it may worsen the dynamic hyperinflation in patients with airflow limitation. [23,24].

Previous in vitro studies in adult and pediatric models reported that jet nebulizer placement in the inspiratory limb farther away from the subject improved aerosol delivery during MV due to the reservoir effect of the ventilator tubing, which accumulates aerosol drugs during ventilation [12,32–34]. In the present study, 39.8% respondents chose to place the nebulizer at the inspiratory limb near the Y-piece, which is the position associated with the lowest drug delivery [35].

The European Society of Clinical Microbiology and Infectious Diseases (ESCMID) recommended the use of specific ventilator settings during nebulization, which included the use of a volume-controlled mode using a constant inspiratory flow, a respiratory rate of 12 to 15 bpm, a tidal volume of 8 mL/kg, an inspiratory: expiratory (I:E) ratio of 50%, an inspiratory pause of 20% and a positive end-expiratory pressure of 5 to 10 cm H2O [25]. These recommendations are used specifically for aerosol antibiotics. In the current study, these recommended ventilator settings were not followed accurately; only 8.4% of the respondents used an inspiratory pause, 15.3% adjusted the PEEP to 5 to 10 cm H2O, 9.2% stopped using the heated humidifier and 14.6% used a constant inspiratory flow. Although the ESCMID recommended the use of a vibrating mesh nebulizer over the ultrasonic and jet nebulizers, only 14.6% respondents used the vibrating mesh nebulizer in the current study. Most respiratory care practitioners prefer to use ultrasonic and jet nebulizers in mainland China. Our results are consistent with another international study, wherein only a minority of the respondents used a vibrating mesh nebulizer (14%) [9]. Thus, the findings of our study indicate that more training programs are needed for respiratory care practitioners in mainland China. Furthermore, less than half of the institutions in this study employed a protocol for aerosol therapy during MV. Therefore, efforts to improve the awareness of standard aerosol therapy practice are warranted. It is the mission of the CURC to establish a practice guideline for the implementation of aerosol therapy in mainland China.

This study has several limitations. First, although the respondents were recruited from all the regions of the country, the response rate was not balanced between the regions. The majority of the respondents were from developed provinces with high GDP (Table 3). Moreover, the majority of the individual questionnaire responses were obtained from tertiary care hospitals (Table 1) indicating a potential bias. Second, the study was based on a questionnaire survey, which was subject to the recall bias. However, we have tried to minimize this bias during the training process. Members were asked to follow the detailed instructions on how to answer the questionnaire, aiming to minimize potential bias and errors. Third, this study was performed in mainland China, and the results may not extrapolate to other countries or regions. Finally, the survey did not link the preferred type of nebulizer with a given pharmacological agent. For example, the use of jet nebulizers for bronchodilators is appropriate as the particles do not need to be very small to reach the bronchi; alternatively, for antibiotics, tiny particles need to be generated by the nebulizers for the medication to reach the alveoli. In such cases, a vibrating mesh plate is required. Nevertheless, this survey was designed based on the idea that a single type of nebulizer is used in hospitals for all kinds of medications.

## Conclusion

In conclusion, the present study examined the implementation of aerosol therapy in mainland China and found that there was substantial heterogeneity among institutions. The implementation of aerosol therapy during MV was not uniformly consistent with recent practice guidelines. Hence, additional efforts by the CURC are needed to improve the implementation of this therapy in this country.

## Supporting information

S1 Dataset Deidentified data for the current analysis. (CSV)

S1 Questionnaire The questionnaire used for the current study. (DOCX)

## Abbreviations

MV: mechanical ventilation
CURC: China Union of Respiratory Care
IMV: invasive mechanical ventilation
NIMV: non-invasive mechanical ventilation
MDI: metered dose inhaler
PEEP: positive end expiratory pressure
LCA: Latent class analysis
BIC: Bayesian information criterion
AIC: Akaike information criterion

## References

[1] Dugernier J, Ehrmann S, Sottiaux T, Roeseler J, Wittebole X, Dugernier T, et al Aerosol delivery during invasive mechanical ventilation: a systematic review. Crit Care. BioMed Central; 2017;21: 264 10.1186/s13054-017-1844-5 29058607 PMC5651640

[2] Mercier E, Darrouzain F, Montharu J, Guillon A, Diot P, Paintaud G, et al Lung and serum teicoplanin concentration after aerosol and intravenous administration in a rat model. J Aerosol Med Pulm Drug Deliv. Mary Ann Liebert, Inc. 140 Huguenot Street, 3rd Floor New Rochelle, NY 10801 USA; 2014;27: 306–312. 10.1089/jamp.2013.1060 24320618

[3] Goldstein I, Wallet F, Nicolas-Robin A, Ferrari F, Marquette C-H, Rouby J-J. Lung deposition and efficiency of nebulized amikacin during Escherichia coli pneumonia in ventilated piglets. Am J Respir Crit Care Med. American Thoracic Society; 2002;166: 1375–1381. 10.1164/rccm.200204-363OC 12406838

[4] Lu Q, Luo R, Bodin L, Yang J, Zahr N, Aubry A, et al Efficacy of high-dose nebulized colistin in ventilator-associated pneumonia caused by multidrug-resistant Pseudomonas aeruginosa and Acinetobacter baumannii. Anesthesiology. The American Society of Anesthesiologists; 2012;117: 1335–1347. 10.1097/ALN.0b013e31827515de 23132092

[5] Solé-Lleonart C, Rouby J-J, Blot S, Poulakou G, Chastre J, Palmer LB, et al Nebulization of Antiinfective Agents in Invasively Mechanically Ventilated Adults: A Systematic Review and Meta-analysis. Anesthesiology. 2017;126: 890–908. 10.1097/ALN.0000000000001570 28248714

[6] van Meenen DMP, van der Hoeven SM, Binnekade JM, de Borgie CAJM, Merkus MP, Bosch FH, et al Effect of On-Demand vs Routine Nebulization of Acetylcysteine With Salbutamol on Ventilator-Free Days in Intensive Care Unit Patients Receiving Invasive Ventilation: A Randomized Clinical Trial. JAMA. American Medical Association; 2018;319: 993–1001. 10.1001/jama.2018.0949 29486489 PMC5885882

[7] Hassan NA, Awdallah FF, Abbassi MM, Sabry NA. Nebulized Versus IV Amikacin as Adjunctive Antibiotic for Hospital and Ventilator-Acquired Pneumonia Postcardiac Surgeries: A Randomized Controlled Trial. Crit Care Med. 2018;46: 45–52. 10.1097/CCM.0000000000002695 28857848

[8] Rello J, Sole-Lleonart C, Rouby JJ, Chastre J, Blot S, Poulakou G, et al Use of nebulized antimicrobials for the treatment of respiratory infections in invasively mechanically ventilated adults: a position paper from the European Society of Clinical Microbiology and Infectious Diseases. Clin Microbiol Infect. 2017;23: 629–639. 10.1016/j.cmi.2017.04.011 28412382

[9] For the REVA research network, Ehrmann S, Roche-Campo F, Sferrazza Papa GF, Isabey D, Brochard L, et al Aerosol therapy during mechanical ventilation: an international survey. Intensive Care Med. Springer-Verlag; 2013;39: 1048–1056. 10.1007/s00134-013-2872-5 23525741

[10] MacIntyre NR, Silver RM, Miller CW, Schuler F, Coleman RE. Aerosol delivery in intubated, mechanically ventilated patients. Crit Care Med. 1985;13: 81–84. 10.1097/00003246-198502000-00005 3967508

[11] Harvey CJ, O'Doherty MJ, Page CJ, Thomas SH, Nunan TO, Treacher DF. Comparison of jet and ultrasonic nebulizer pulmonary aerosol deposition during mechanical ventilation. Eur Respir J. 1997;10: 905–909. 9150333

[12] Ari A, Atalay OT, Harwood R, Sheard MM, Aljamhan EA, Fink JB. Influence of nebulizer type, position, and bias flow on aerosol drug delivery in simulated pediatric and adult lung models during mechanical ventilation. Respir Care. 2010;55: 845–851. 20587095

[13] Berlinski A, Willis JR. Effect of Tidal Volume and Nebulizer Type and Position on Albuterol Delivery in a Pediatric Model of Mechanical Ventilation. Respir Care. Respiratory Care; 2015;60: 1424–1430. 10.4187/respcare.04013 25969513

[14] Dhand R. Aerosol delivery during mechanical ventilation: from basic techniques to new devices. J Aerosol Med Pulm Drug Deliv. Mary Ann Liebert, Inc. 2 Madison Avenue Larchmont, NY 10538 USA; 2008;21: 45–60. 10.1089/jamp.2007.0663 18518831

[15] Dolovich MB, Ahrens RC, Hess DR, Anderson P, Dhand R, Rau JL, et al Device selection and outcomes of aerosol therapy: Evidence-based guidelines: American College of Chest Physicians/American College of Asthma, Allergy, and Immunology. Chest. 2005;127: 335–371. 10.1378/chest.127.1.335 15654001

[16] Li J, Ni Y, Tu M, Ni J, Ge H, Shi Y, et al Respiratory Care Education and Clinical Practice in Mainland China. Respir Care. 2018;63: 1239–1245. 10.4187/respcare.06217 30042123

[17] Zhang Z. Univariate description and bivariate statistical inference: the first step delving into data. Ann Transl Med. 2016;4: 91–91. 10.21037/atm.2016.02.11 27047950 PMC4791343

[18] Zhang Z, Gayle AA, Wang J, Zhang H, Cardinal-Fernández P. Comparing baseline characteristics between groups: an introduction to the CBCgrps package. Ann Transl Med. 2017;5: 484–484. 10.21037/atm.2017.09.39 29299446 PMC5750271

[19] Rindskopf D, Rindskopf W. The value of latent class analysis in medical diagnosis. Stat Med. Wiley Subscription Services, Inc., A Wiley Company; 1986;5: 21–27. 10.1002/sim.4780050105 3961312

[20] Collins LM, Lanza ST. Latent Class and Latent Transition Analysis: With Applications in the Social, Behavioral, and Health Sciences. Latent Class and Latent Transition Analysis: With Applications in the Social, Behavioral, and Health Sciences. Hoboken, NJ, USA: John Wiley & Sons, Inc; 2010 pp. 1–295. 10.1002/9780470567333

[21] Nylund KL, Asparouhov T, Muthén BO. Deciding on the number of classes in latent class analysis and growth mixture modeling: A Monte Carlo simulation study. Structural Equation Modeling. 4 ed. 2007;14: 535–569. 10.1080/10705510701575396

[22] Zhang Z, Abarda A, Contractor AA, Wang J, Dayton CM. Exploring heterogeneity in clinical trials with latent class analysis. Ann Transl Med. 2018;6: 119–119. 10.21037/atm.2018.01.24 29955579 PMC6015948

[23] Reychler G, Leal T, Aubriot A-S, Liistro G. In Vitro and In Vivo Evaluation of the Combination of Oscillating Positive Expiratory Pressure and Nebulization: A Randomized Cross-Over Study. Arch Bronconeumol. 2017;53: 695–697. 10.1016/j.arbres.2017.04.011 28558927

[24] Maccari JG, Teixeira C, Savi A, de Oliveira RP, Machado AS, Tonietto TF, et al Nebulization during spontaneous breathing, CPAP, and bi-level positive-pressure ventilation: a randomized analysis of pulmonary radioaerosol deposition. Respir Care. Respiratory Care; 2014;59: 479–484. 10.4187/respcare.02518 24003242

[25] Rello J, Rouby JJ, Sole-Lleonart C, Chastre J, Blot S, Luyt CE, et al Key considerations on nebulization of antimicrobial agents to mechanically ventilated patients. Clin Microbiol Infect. 2017;23: 640–646. 10.1016/j.cmi.2017.03.018 28347790

[26] Sole-Lleonart C, Roberts JA, Chastre J, Poulakou G, Palmer LB, Blot S, et al Global survey on nebulization of antimicrobial agents in mechanically ventilated patients: a call for international guidelines. Clin Microbiol Infect. 2016;22: 359–364. 10.1016/j.cmi.2015.12.016 26723563

[27] Solé-Lleonart C, Rouby J-J, Chastre J, Poulakou G, Palmer LB, Blot S, et al Intratracheal Administration of Antimicrobial Agents in Mechanically Ventilated Adults: An International Survey on Delivery Practices and Safety. Respir Care. Respiratory Care; 2016;61: 1008–1014. 10.4187/respcare.04519 26957647

[28] Alves J, Alp E, Koulenti D, Zhang Z, Ehrmann S, Blot S, et al Nebulization of antimicrobial agents in mechanically ventilated adults in 2017: an international cross-sectional survey. Eur J Clin Microbiol Infect Dis. Springer Berlin Heidelberg; 2018;37: 785–794. 10.1007/s10096-017-3175-5 29318460

[29] Ari A. Aerosol Therapy in Pulmonary Critical Care. Respir Care. Respiratory Care; 2015;60: 858–74– discussion 874–9. 10.4187/respcare.03790 26070580

[30] Ge H-Q, Wang J-M, Lin H-L, Fink JB, Luo R, Xu P, et al Effect of Nebulizer Location and Spontaneous Breathing on Aerosol Delivery During Airway Pressure Release Ventilation in Bench Testing. J Aerosol Med Pulm Drug Deliv. Mary Ann Liebert, Inc., publishers 140 Huguenot Street, 3rd Floor New Rochelle, NY 10801 USA; 2019;32: 34–39. 10.1089/jamp.2018.1457 30199313

[31] Guérin C, Fassier T, Bayle F, Lemasson S, Richard J-C. Inhaled bronchodilator administration during mechanical ventilation: how to optimize it, and for which clinical benefit? J Aerosol Med Pulm Drug Deliv. Mary Ann Liebert, Inc. 2 Madison Avenue Larchmont, NY 10538 USA; 2008;21: 85–96. 10.1089/jamp.2007.0630 18518835

[32] Berlinski A, Willis JR. Albuterol delivery by 4 different nebulizers placed in 4 different positions in a pediatric ventilator in vitro model. Respir Care. Respiratory Care; 2013;58: 1124–1133. 10.4187/respcare.02074 23107173

[33] Moraine J-J, Truflandier K, Vandenbergen N, Berré J, Mélot C, Vincent J-L. Placement of the nebulizer before the humidifier during mechanical ventilation: Effect on aerosol delivery. Heart Lung. 2009;38: 435–439. 10.1016/j.hrtlng.2008.12.005 19755194

[34] Anderson AC, Dubosky MN, Fiorino KA, Quintana V, Kaplan CA, Vines DL. The Effect of Nebulizer Position on Aerosolized Epoprostenol Delivery in an Adult Lung Model. Respir Care. Respiratory Care; 2017;62: 1387–1395. 10.4187/respcare.05344 28720675

[35] Ballard J, Lugo RA, Salyer JW. A survey of albuterol administration practices in intubated patients in the neonatal intensive care unit. Respir Care. 2002;47: 31–38. 11749685

