# Supplementary material for: Practice pattern of aerosol therapy among patients undergoing mechanical ventilation in mainland China: A web-based survey involving 447 hospitals
Source: PLoS One. 2019 Aug 29;14(8):e0221577. doi: 10.1371/journal.pone.0221577 (PMC6715194; doi:10.1371/journal.pone.0221577)
Supplement: S1 Questionnaire — (DOCX) [file pone.0221577.s002.docx]

Questionnaire of the aerosol therapy for mechanical ventilation in mainland China

# Basic information for respondents

Name (initials):

Phone number:

Email:

Province:

Level of your Hospital:

Name of your hospital:

Number of beds of your hospital:

Your department:

How long have you been in clinical practice?

Your specialty:

infectious disease/microbiology;

critical care/anesthesiology;

internal medicine/pulmonary medicine;

respiratory care;

surgery;

nurse/pharmacist;

others

How many patients received mechanical ventilation in the past one month in your department?

What’s your overall feeling for the effectiveness of aerosol therapy?

Excellent

General

Unsatisfactory

Poor

Have you ever participated in such kind of survey in the past?

Yes

No

Have you ever been using aerosol therapy for patients with invasive mechanical ventilation?

Yes

No

Have you ever been using aerosol therapy for patients with non-invasive mechanical ventilation?

Yes

No

What’s the drug did you use for aerosol therapy (multiple choice)?

Vasodilators such as terbutaline and salbutamol

Antibiotics

Mucolytic agent

Topical corticosteroids

Systemic corticosteroids

Others

What type of nebulizer did you use?

| Ultrasonic nebulizer |
| --- |
| Jet nebulizer |
| vibrating-mesh nebulizer |
| Metered dose inhaler |
| Others |

| What type of jet nebulizer did you use? |
| --- |
| External gas source |
| External nebulizer pump |
| Nebulizer within ventilator |
| Others |

| What is the position of nebulizer for small-volume nebulizer? |
| --- |
| Inspiratory limb near Y-piece |
| Humidifier proximal to ventilator |
| Humidifier proximal to patient |
| Others |

| What is the position of nebulizer for metered dose nebulization? |
| --- |
| Inspiratory limb near Y-piece |
| Humidifier proximal to ventilator |
| Humidifier proximal to patient |
| Others |

| Did you use holding chambers/spacers for metered dose nebulization? |
| --- |
| Yes |
| No |

| How often did your institution change the filter at expiratory circuit? |
| --- |
| Every time after nebulization |
| Once daily |
| Twice a week |
| Once a week |
| More than once a week |

| Did you Change ventilator parameters during nebulization? |
| --- |
| Never change |
| Increase PEEP |
| Decrease inspiratory flow |
| Use constant inspiratory flow |
| Increase inspiratory time |
| Use inspiratory pause |
| Increase tidal volume |
| Stop heated humidifier |
| Place filter on expiratory circuit |
| Sedation to avoid dyssynchrony |
| Use base flow rate |
| Others |

| What is the PEEP level during nebulization (cmH2O)? |
| --- |
| 0 |
| 1-5 |
| 6-10 |
| >10 |
| PEEP not changed |

| Did you use any of the following specific mode during nebulization? |
| --- |
| Pressure control |
| Pressure support |
| Volume control |
| SIMV |
| High-frequency ventilation |
| Others |

| Did you use nebulization protocol in your institution? |
| --- |
| Yes |
| No |

| How did you assess the effectiveness of nebulization? |
| --- |
| Waveform |
| Breathing sound |
| Inspection of breathing appearance |
| Not assess routinely |
| Others |
